# *CDKN2A* deletion in supratentorial ependymoma with *RELA* alteration indicates a dismal prognosis: a retrospective analysis of the HIT ependymoma trial cohort

**DOI:** 10.1007/s00401-020-02169-z

**Published:** 2020-06-08

**Authors:** Stephanie T. Jünger, Felipe Andreiuolo, Martin Mynarek, Inken Wohlers, Sven Rahmann, Ludger Klein-Hitpass, Evelyn Dörner, Anja zur Mühlen, Natalia Velez-Char, Katja von Hoff, Monika Warmuth-Metz, Rolf-Dieter Kortmann, Beate Timmermann, Andre von Bueren, Stefan Rutkowski, Torsten Pietsch

**Affiliations:** 1 Department of Neuropathology, Institute of Neuropathology, DGNN Brain Tumor Reference Center, University of Bonn, Venusberg-Campus 1, 53127 Bonn, Germany; 2 Department of Pediatric Hematology and Oncology, University Hospital Hamburg-Eppendorf, Hamburg, Germany; 3 Genome Informatics, Institute of Human Genetics, University of Duisburg-Essen, Essen, Germany; 4 Department of Cell Biology (Tumor Research), University of Duisburg-Essen, Essen, Germany; 5 Institute of Diagnostic and Interventional Neuroradiology, University Hospital Würzburg, Würzburg, Germany; 6 Department of Radiation Oncology, University Hospital Leipzig, Leipzig, Germany; 7 Westdeutsches Protonentherapiezentrum, Essen, Germany; 8 Present Address: Department of Neurosurgery, University of Cologne Medical Center, Cologne, Germany; 9 Present Address: Medical Systems Biology Division, Lübeck Institute of Experimental Dermatology and Institute for Cardiogenetics, University of Lübeck, Lübeck, Germany; 10 Present Address: Division of Pediatric Hematology and Oncology, Department of Pediatrics, Obstetrics and Gynecology, University Hospital of Geneva, Geneva, Switzerland

Pediatric supratentorial ependymomas with *RELA* fusions (RELA-EP) have been identified as a unique novel tumor entity [9, 10]. Fusions between *C11orf95* and *RELA* pathologically activate the NFκB signaling pathway indicated by nuclear accumulation of p65-RelA. Deletions of *CDKN2A* encoding the negative cell-cycle regulator p16 have been described in a subset of supratentorial ependymomas, associated with worse outcome [2, 5, 7]. We assessed the frequency and prognostic impact of *CDKN2A* deletions in a cohort of 54 RELA-EP in children treated according to HIT2000-E protocols (for detailed demographic information, see supplementary materials and methods and supplementary table 1).

High-resolution, genome-wide copy number profiles were generated by molecular inversion probe (MIP) assay. Chromosomal breaks were identified within the *C11orf95* and *RELA* genes corresponding to fusion transcripts (Fig. 1a, d). All cases showed pathological nuclear accumulation of p65-RelA as a hallmark of RELA-EP (Fig. 1f). Homozygous deletion (complete loss) of *CDKN2A* was detected in 9 of 54 (16.7%) cases (Fig. 1c); and 8 of these (88.9%) showed a concordant complete loss of p16 protein (Fig. 1g). In one case, few tumor cells expressed p16 protein indicating retained *CDKN2A* alleles in single cells. Fourteen cases (25.9%) harbored a hemizygous deletion of *CDKN2A.* In these, p16 protein was retained in 92.9% of cases tested—one case lacked p16 protein expression indicating the inactivation of the second allele by alternative mechanisms. Thirty-one of 54 cases (57.4%) had no deletion of *CDKN2A;* all showed p16 protein expression (Fig. 1h). Immunohistochemistry for p16, therefore, may serve as a surrogate for complete *CDKN2A* loss, but cannot differentiate between hemizygous and wild-type status. There was no statistical association between *CDKN2A* deletions and mitotic activity as previously described in *IDH*-mutant glioma [1]. The presence of *CDKN2A* deletions (homo- or hemizygous) correlated with higher age at diagnosis in line with the literature [3, 5, 8]. *CDKN2A* deletion may also occur as secondary event in tumor progression [7].

**Fig. 1:**
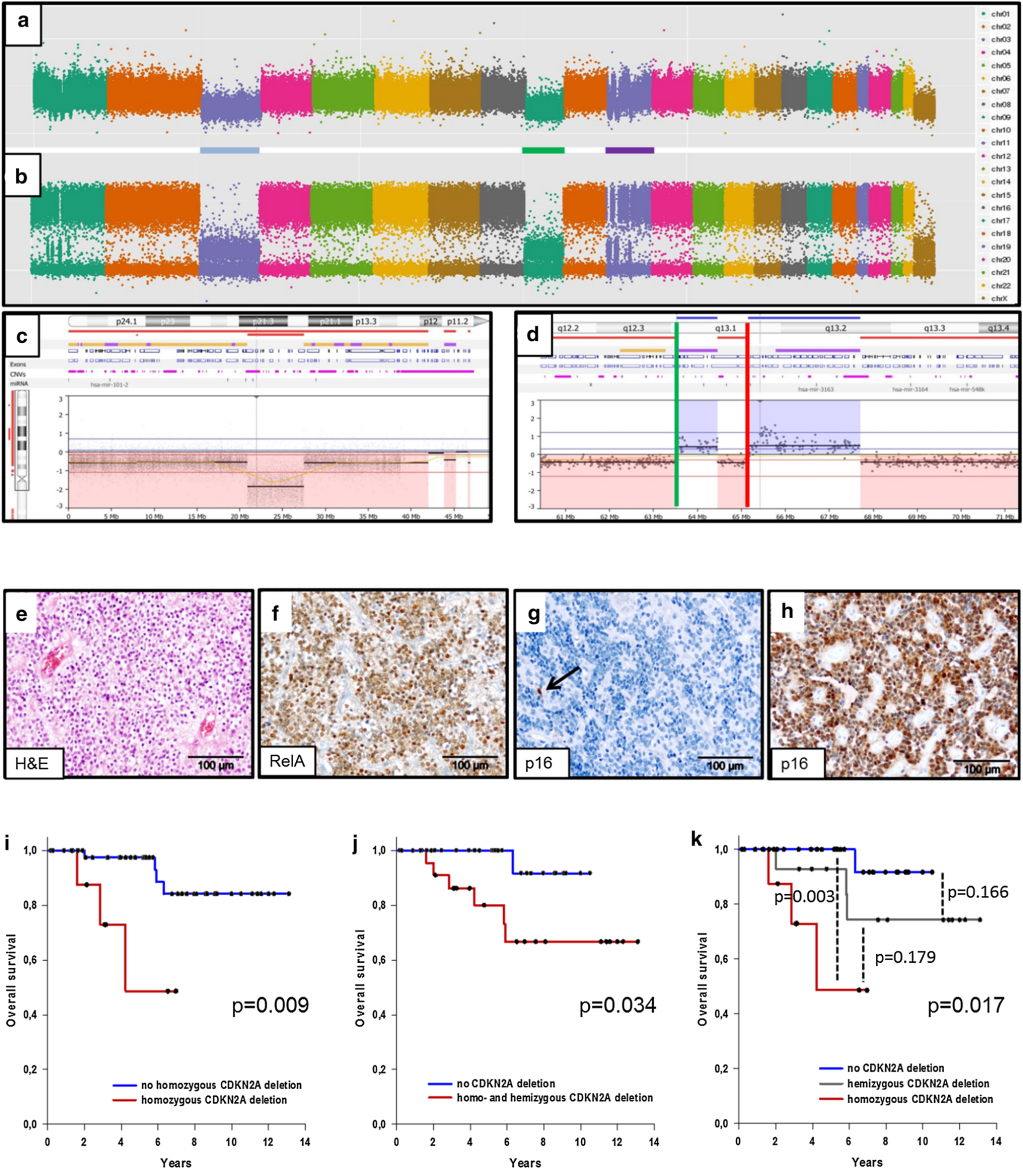
**a** Genomic copy number profile and **b** allele distribution  (MIP) of a RELA-EP showing chromothripsis of chromosome 11; **c** case with homozygous *CDKN2A* deletion; **d** case showing breaks in *C11orf95* (green bar) and *RELA* (red bar); **e** clear cell morphology; **f** nuclear p65-RelA; **g** case with homozygous *CDKN2A* deletion/loss of p16 protein (arrow, endothelial cell as internal positive control); **h** case without *CDKN2A* deletion/retained p16; **i**–**k** Kaplan–Meier analysis, impact of *CDKN2A* deletions on OS

To identify possible differences between RELA-EP with versus without *CDKN2A* deletion on the transcript level, 12 RELA-EP were analyzed by RNA sequencing for differentially expressed genes. After correction for multiple testing, five genes were found significantly downregulated including *CDKN2A* and *CDKN2B* and their neighboring gene *MTAP* (*S*-methyl-5′-thioadenosine phosphorylase) located in the deleted region. MTAP is a key enzyme in the methionine salvage pathway. Its deletion leads to dependence on the activity of the methyltransferase PRMT5 [6] which can be blocked by PRMT5 inhibitors as interesting novel therapeutics in *MTAP* deleted tumors. In addition, *KIF7* (15q26) encoding a cilia-associated protein and *ZNF536* (19q12) encoding a neuronal marker were found downregulated. *GABRA2* (4p12) encoding the gamma-aminobutyric acid receptor subunit alpha-2 was found highly upregulated in *CDKN2A* deleted tumors (supplementary figure 3).

Kaplan–Meier analysis revealed a significant correlation between *CDKN2A* deletions and overall survival status (OS). Different groups were compared: (1) homozygous *CDKN2A* deletion vs. hemizygous *CDKN2A* deletion and tumors with two retained alleles (*p* = 0.009); (2) homo- or hemizygous *CDKN2A* deletions vs. tumors with two retained alleles (*p* = 0.034) and (3) all three strata separately (*p* = 0.017) (Fig. 1i–k). In contrast to Korshunov et al. [5], neither homozygous nor hemizygous deletion showed prognostic relevance regarding EFS (supplementary figure 2). Predominant clear cell morphology as a histological feature was a favorable prognosticator for OS (*p* = 0.039), and high mitotic activity (> 17 mitoses/10HPF) was a predictor for tumor relapse (*p* = 0.004) as well as OS (*p* = 0.007) (supplementary figure 1). Multivariate analysis confirmed mitotic activity as independent prognostic indicator for EFS (supplementary table 2).

Our data show that deletions of *CDKN2A* represent an objective parameter for risk stratification in RELA-EP. Molecular inversion probe methodology turned out to represent a sensitive and quantitative tool for *CDKN2A* assessment in FFPE material. Apart from ependymoma, homozygous deletions of *CDKN2A* have recently been described as adverse prognostic marker for other CNS tumors, including anaplastic *IDH*-mutant gliomas and *BRAF*-mutant low-grade gliomas [1, 4, 11]. The deletion/inactivation of *CDKN2A* may result in a pathological activation of cyclin-dependent kinases 4/6 targetable by specific inhibitors such as palbociclib. Therefore, *CDKN2A* inactivation in RELA-ependymomas may represent a potential therapeutical target.

## Electronic supplementary material

Below is the link to the electronic supplementary material.

Supplementary table 1, demographics, clinical, neuropathological and genetic information of the patient cohort (DOCX 17 kb) 

Supplementary table 2, multivariate analysis (DOCX 20 kb)

Supplementary figure 1, Kaplan-Meier analysis of age at diagnosis, clear cell morphology and mitotic activity (PPTX 132 kb)

Supplementary figure 2, KM analysis of *CDKN2A* deletion in RELA ependymomas (EFS) (PPTX 116 kb)

Supplementary figure 3, differential expression (RNA sequencing) (PPTX 79 kb)

supplementary materialsand methods (DOCX 20 kb)
