# Supplementary material for: CDKN2A deletion in supratentorial ependymoma with RELA alteration indicates a dismal prognosis: a retrospective analysis of the HIT ependymoma trial cohort
Source: Acta Neuropathol. 2020 Jun 8;140(3):405–7. doi: 10.1007/s00401-020-02169-z (PMC7423858; doi:10.1007/s00401-020-02169-z)
Supplement: Supplementary file 1 — Supplementary table 1, demographics, clinical, neuropathological and genetic information of the patient cohort (DOCX 17 kb) [file 401_2020_2169_MOESM1_ESM.docx]

**Supplementary Table 1. Demographic, histological, genetic and clinical parameters of the cohort of patients with RELA ependymomas.**
