# Supplementary material for: CDKN2A deletion in supratentorial ependymoma with RELA alteration indicates a dismal prognosis: a retrospective analysis of the HIT ependymoma trial cohort
Source: Acta Neuropathol. 2020 Jun 8;140(3):405–7. doi: 10.1007/s00401-020-02169-z (PMC7423858; doi:10.1007/s00401-020-02169-z)
Supplement: Supplementary file 2 — Supplementary table 2, multivariate analysis (DOCX 20 kb) [file 401_2020_2169_MOESM2_ESM.docx]

**Materials and Methods**

**Patients**

Ependymomas of 57 children below the age of 19 years were retrieved from the archives of the Institute of Neuropathology, University of Bonn Medical Center (Bonn, Germany) and of the DGNN German Brain Tumor Reference Center (Bonn, Germany). Corresponding demographic and clinical data were provided by the HIT study center at the University of Hamburg Medical Center, Hamburg-Eppendorf, Germany. Informed consent was given at study inclusion by the parents or adolescent patients themselves. The patients received risk-adapted treatment according to guidelines of the HIT- ependymoma protocols with a combination of radio- and chemotherapy adjusted to age at diagnosis, WHO grading and extent of resection. Between 2001 and 2010, the patients were enrolled into the HIT2000 ependymoma trial (ClinicalTrials.gov NCT00303810). Two patients of our cohort were diagnosed between 12/1999 and 7/2000 before the official start of the study but treated according the same strategy. This included modified HIT-SKK chemotherapy (without intraventricular methotrexate) and irradiation, which was postponed until after the age of 18 months if possible. In case of residual tumor, patients were evaluated for potential second-look surgery after each therapy element.

**Histopathology and immunohistochemistry**

All tumors were centrally reviewed at the DGNN German Brain Tumor Reference Center (Bonn, Germany) and re-classified according to the revised WHO classification of tumors of the CNS 2016 [5]. Mitotic activity was assessed in 4µm thick H&E-stained FFPE slides by counting mitotic figures in ten high power fields (HPF; area 0.238 mm^2^). Presence of more than 17 mitoses/10 HPF (median) was defined as high mitotic activity. Further histological features including necrosis, vascular proliferation and clear cell morphology were also assessed. Immunohistochemical staining - performed on an immunostaining system (BenchMark XT, Ventana-Roche, Mannheim, Germany) - included Ki67 (MAb MIB-1; Dako, Glostrup, Denmark), phosphohistone-H3 (Biocare, Concord, USA), p16 protein (MAb E6H4, Ventana-Roche, Darmstadt, Germany), epithelial membrane antigen (EMA; Mab 29, Dako) and p65-RelA (rabbit antibody D14E12, Cell Signaling, Danvers, U.S.A.).

**DNA extraction and copy number analysis**

On H&E-stained FFPE sections representative areas with at least 80 % tumor cell content were identified for microdissection and DNA extraction from serial sections using the QIAamp DNA Mini Kit (Qiagen, Hilden, Germany) according to the manufacturer’s instructions. In order to identify genome-wide copy number alterations and allelic imbalances we used molecular inversion probe assays (OncoScan FFPE express 330K arrays, versions 2 and 3; Affymetrix/ ThermoFisher, Santa Clara, CA, US) as previously described [1, 3]. In cases where fresh frozen material was available DNA-extraction was performed after histological review of frozen sections by phenol-chloroform-method with ethanol-precipitation [7]. Afterward the DNA was analyzed using genome-wide human SNP array 6.0 (Affymetrix).

Raw data were further analyzed with the Nexus Copy Number software, version 7.5 (BioDiscovery, El Segundo, CA, USA). DNA from FFPE cerebellar tissue served as normal control. The SNP-FASST-2 segmentation algorithm was used for data processing. All cases were reviewed individually and, if necessary diploidy correction was performed manually. Individual chromosomal alterations were identified and tumors were assigned to one of three previously described genomic phenotypes; tumors with alterations of whole chromosomes to the “numerical”, those lacking alterations to the “balanced” and tumors with partial alterations of chromosomes to the “structural” phenotype [4].

**RNA extraction and detection of *C11orf95-RELA and YAP1-MAMLD1* fusions**

The tumors were further tested for presence of *C11orf95-RELA* and *YAP1-MAMLD1* fusions. RNA was extracted from FFPE or fresh frozen tissue, followed by RT-PCR and Sanger sequencing, as described previously [1, 2, 6].

**RNA sequencing and identification of differentially expressed genes**

From 12 fresh-frozen RELA ependymomas (5 cases with homozygous *CDKN2A* loss, 7 cases without loss), RNA of high quality could be extracted and RNA sequencing was performed as previously described [6]. Differential gene expression was determined using a workflow for RNA-seq differential expression analysis based on Kallisto and Sleuth (<https://github.com/snakemake-workflows/rna-seq-kallisto-sleuth>). This workflow consists of adapter removal, transcript quantification and statistical estimation of significance, including multiple testing correction, and estimation of effect size of differential expression. To analyze potentially affected pathways, the Enrichr platform was used (<https://amp.pharm.mssm.edu/Enrichr>) which allows the analysis of multiple gene-set libraries.

**Statistical analyses**

Using IBM SPSS statistics version 25 (IBM, Bonn, Germany), categorial and continuous variables were compared with two tailed Fischer’s exact test or Mann-Whitney U-Test respectively. Survival distributions for EFS and OS were evaluated by Kaplan-Meier plots applying log-rank (Mantel-Cox) method. Results with a p-value <0.05 were regarded as significant.

**References to supplementary Materials and Methods**

1. Andreiuolo F, Varlet P, Tauziède-Espariat A et al (2019) Childhood supratentorial ependymomas with *YAP1-MAMLD1* fusion: an entity with characteristic clinical, radiological, cytogenetic and histopathological features. Brain Pathol 29:205-216

2. Figarella-Branger D, Lechapt-Zalcman E, Tabouret E et al (2016) Supratentorial clear cell ependymomas with branching capillaries demonstrate characteristic clinicopathological features and pathological activation of nuclear factor-kappaB signaling. Neuro-Oncol 18:919–927

3. Japp AS, Gessi M, Messing-Jünger M et al (2015) High-Resolution Genomic Analysis Does Not Qualify Atypical Plexus Papilloma as a Separate Entity Among Choroid Plexus Tumors. J Neuropathol Exp Neurol 74:110–120

4. Jünger ST, Mynarek M, Wohlers I et al (2019) Improved risk-stratification for posterior fossa ependymoma of childhood considering clinical, histological and genetic features - a retrospective analysis of the HIT ependymoma trial cohort. Acta Neuropathol Commun 7:181

5. Louis et. al (2016) WHO classification of tumours of the central nervous system, Revised 4th edition. International Agency for Research on Cancer, Lyon

6. Pietsch T, Wohlers I, Goschzik T et al (2014) Supratentorial ependymomas of childhood carry C11orf95–RELA fusions leading to pathological activation of the NF-κB signaling pathway. Acta Neuropathol 127:609–611

7. Sambrook J, Fritsch E, Maniatis T (1989) Molecular cloning: A laboratory manual: Vol. 2, 2. ed. Cold Spring Harbor, S.l.
