# Supplementary material for: CDKN2A deletion in supratentorial ependymoma with RELA alteration indicates a dismal prognosis: a retrospective analysis of the HIT ependymoma trial cohort
Source: Acta Neuropathol. 2020 Jun 8;140(3):405–7. doi: 10.1007/s00401-020-02169-z (PMC7423858; doi:10.1007/s00401-020-02169-z)
Supplement: Supplementary file 3 — Supplementary figure 1, Kaplan-Meier analysis of age at diagnosis, clear cell morphology and mitotic activity (PPTX 132 kb) [file 401_2020_2169_MOESM3_ESM.pptx]

## Slide 1
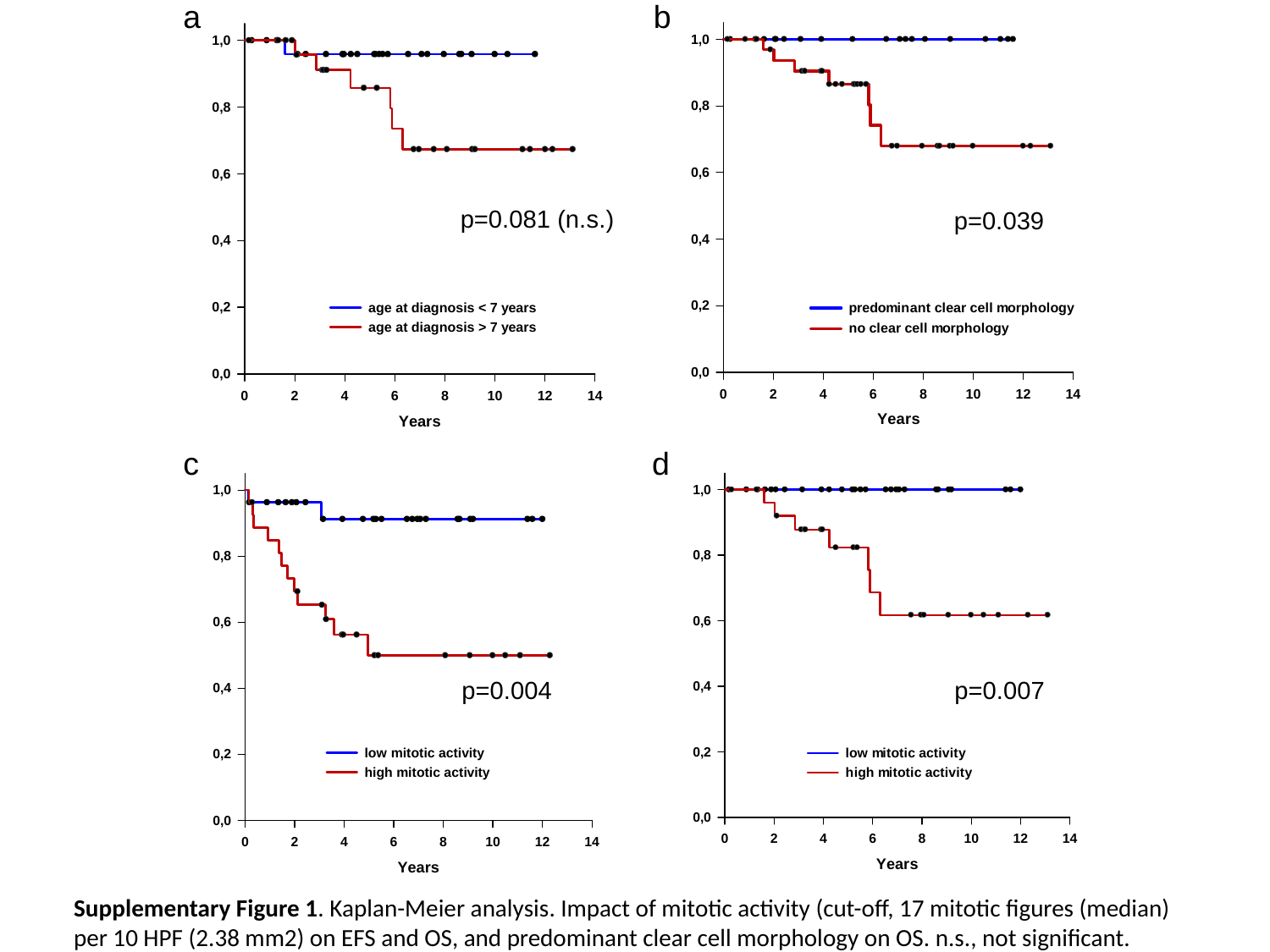

a
b
p=0.081 (n.s.)
p=0.039
c
d
p=0.004
p=0.007
Supplementary Figure 1. Kaplan-Meier analysis. Impact of mitotic activity (cut-off, 17 mitotic figures (median)
per 10 HPF (2.38 mm2) on EFS and OS, and predominant clear cell morphology on OS. n.s., not significant.
