# Supplementary material for: CDKN2A deletion in supratentorial ependymoma with RELA alteration indicates a dismal prognosis: a retrospective analysis of the HIT ependymoma trial cohort
Source: Acta Neuropathol. 2020 Jun 8;140(3):405–7. doi: 10.1007/s00401-020-02169-z (PMC7423858; doi:10.1007/s00401-020-02169-z)
Supplement: Supplementary file 4 — Supplementary figure 2, KM analysis of CDKN2A deletion in RELA ependymomas (EFS) (PPTX 116 kb) [file 401_2020_2169_MOESM4_ESM.pptx]

## Slide 1
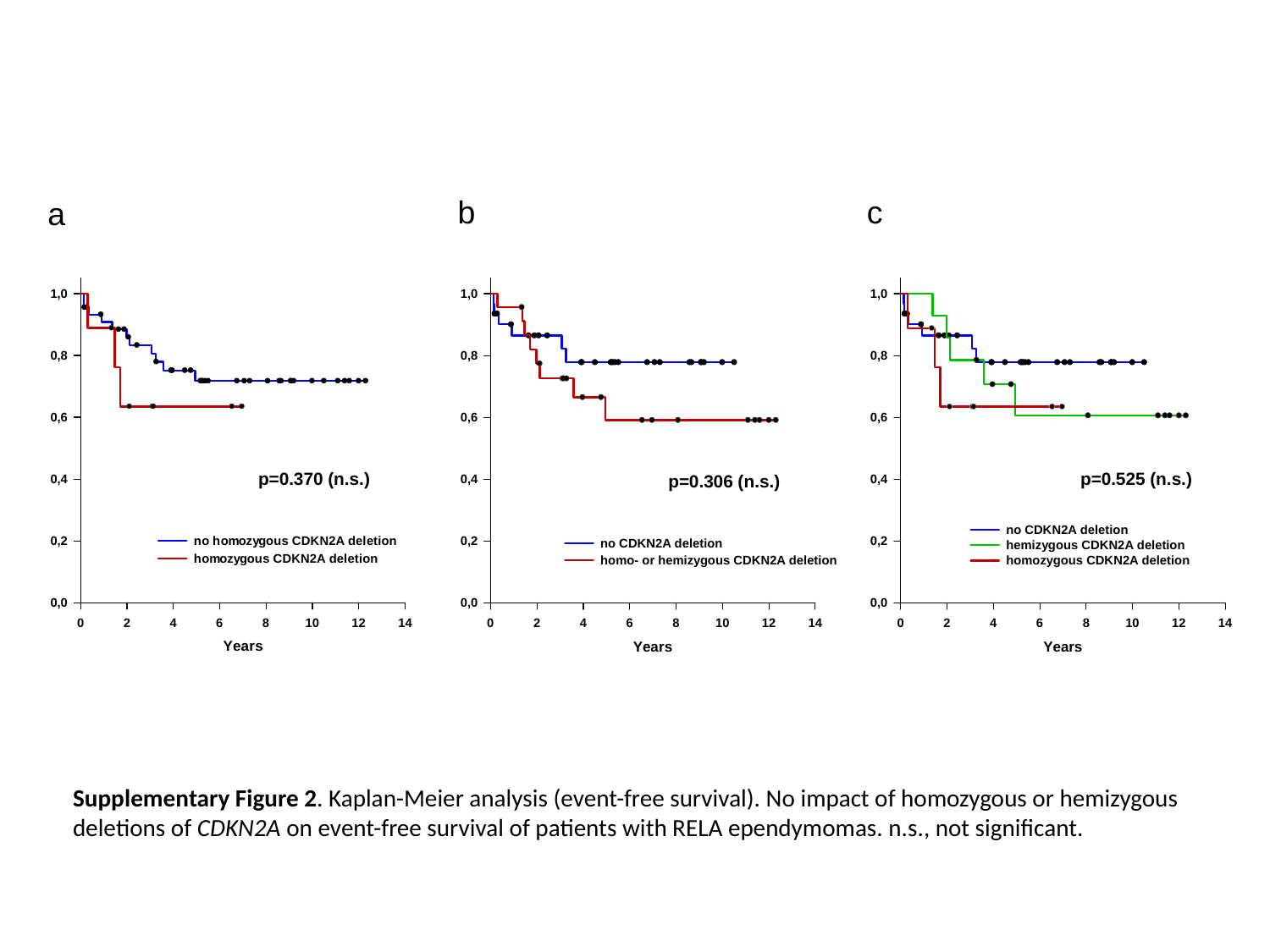

b
c
a
d
p=0.370 (n.s.)
p=0.525 (n.s.)
p=0.306 (n.s.)
Supplementary Figure 2. Kaplan-Meier analysis (event-free survival). No impact of homozygous or hemizygous
deletions of CDKN2A on event-free survival of patients with RELA ependymomas. n.s., not significant.
