# Supplementary material for: CDKN2A deletion in supratentorial ependymoma with RELA alteration indicates a dismal prognosis: a retrospective analysis of the HIT ependymoma trial cohort
Source: Acta Neuropathol. 2020 Jun 8;140(3):405–7. doi: 10.1007/s00401-020-02169-z (PMC7423858; doi:10.1007/s00401-020-02169-z)
Supplement: Supplementary file 5 — Supplementary figure 3, differential expression (RNA sequencing) (PPTX 79 kb) [file 401_2020_2169_MOESM5_ESM.pptx]

## Slide 1
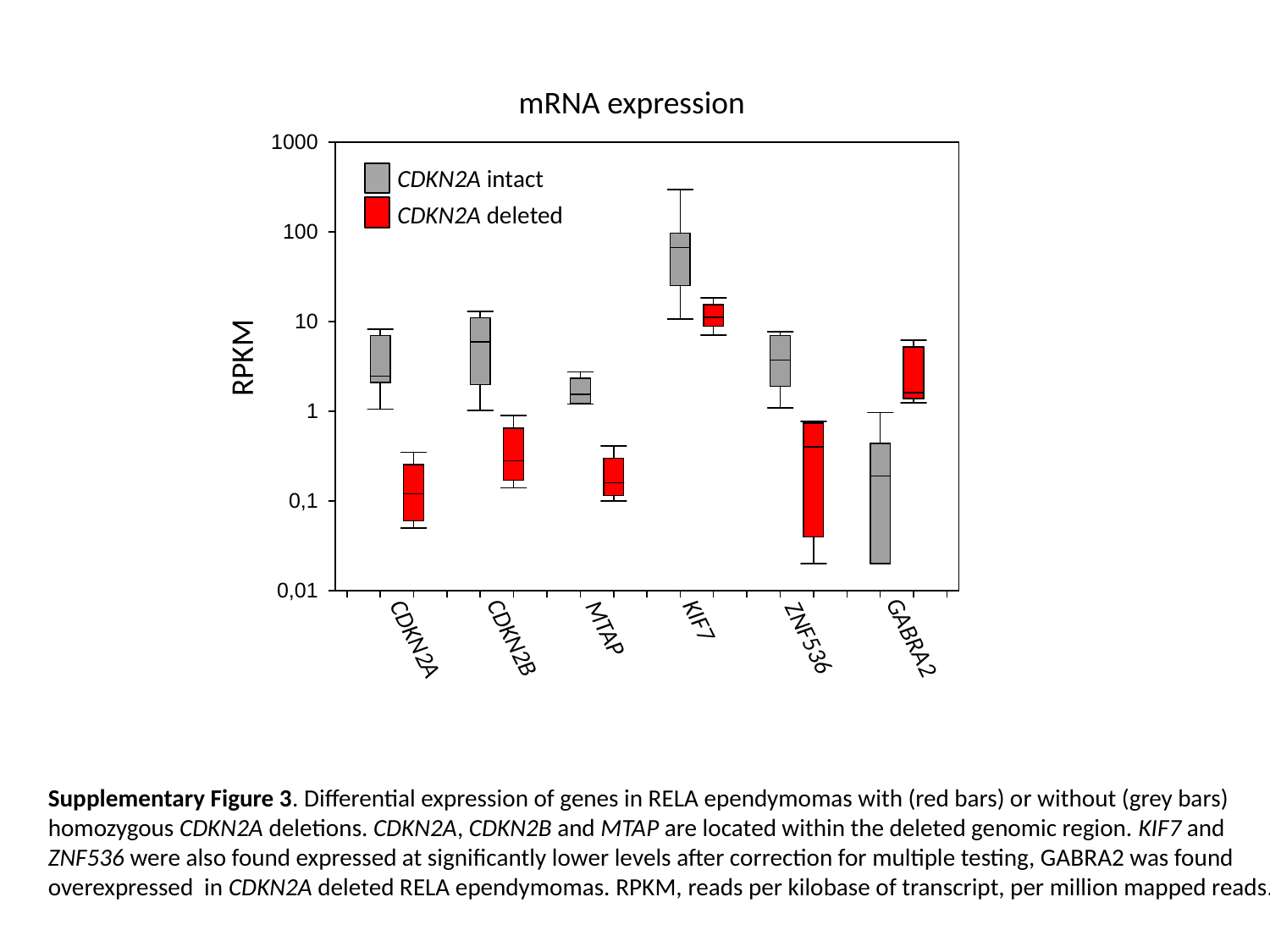

mRNA expression
CDKN2A intact
CDKN2A deleted
RPKM
KIF7
MTAP
ZNF536
GABRA2
CDKN2B
CDKN2A
Supplementary Figure 3. Differential expression of genes in RELA ependymomas with (red bars) or without (grey bars)
homozygous CDKN2A deletions. CDKN2A, CDKN2B and MTAP are located within the deleted genomic region. KIF7 and
ZNF536 were also found expressed at significantly lower levels after correction for multiple testing, GABRA2 was found
overexpressed in CDKN2A deleted RELA ependymomas. RPKM, reads per kilobase of transcript, per million mapped reads.
